# Integrating network analysis with differential expression to uncover therapeutic and prognostic biomarkers in esophageal squamous cell carcinoma

**DOI:** 10.3389/fmolb.2024.1425422

**Published:** 2024-08-21

**Authors:** Sana Khurshid, Shahabuddin Usmani, Raiyan Ali, Saira Hamid, Tariq Masoodi, Hana Q. Sadida, Ikhlak Ahmed, Mohd Shahnawaz Khan, Inara Abeer, Ibrahim Altedlawi Albalawi, Ruqaiah I. Bedaiwi, Rashid Mir, Ammira S. Al-Shabeeb Akil, Ajaz A. Bhat, Muzafar A. Macha

**Affiliations:** ^1^ Watson-Crick Centre for Molecular Medicine, Islamic University of Science and Technology, Awantipora, India; ^2^ Department of Human Genetics-Precision Medicine in Diabetes, Obesity and Cancer Program, Sidra Medicine, Doha, Qatar; ^3^ Council of Scientific and Industrial Research-Institute of Genomics and Integrative Biology, Delhi, India; ^4^ Human Immunology Department, Research Branch, Sidra Medicine, Doha, Qatar; ^5^ Department of Biochemistry, College of Sciences, King Saud University, Riyadh, Saudi Arabia; ^6^ Department of Pathology, Sker-I-Kashmir Institute of Medical Sciences, Srinagar, India; ^7^ Department of Surgical Oncology, Faculty of Medicine, University of Tabuk, Tabuk, Saudi Arabia; ^8^ Faculty of Applied Medical Sciences, Medical Laboratory Technology, University of Tabuk, Tabuk, Saudi Arabia

**Keywords:** esophageal squamous cell carcinoma, the Cancer Genome Atlas, differentially expressed genes, protein-protein interaction, network analysis

## Abstract

**Introduction:** Esophageal squamous cell carcinoma (ESCC) accounts for over 90% of all esophageal tumors. However, the molecular mechanism underlying ESCC development and prognosis remains unclear, and there are still no effective molecular biomarkers for diagnosing or predicting the clinical outcome of patients with ESCC. Here, we used bioinformatics analysis to identify potential biomarkers and therapeutic targets for ESCC.

**Methodology:** Differentially expressed genes (DEGs) between ESCC and normal esophageal tissue samples were obtained by comprehensively analyzing publicly available RNA-seq datasets from the TCGA and GTEX. Gene Ontology (GO) annotation and Reactome pathway analysis identified the biological roles of the DEGs. Moreover, the Cytoscape 3.10.1 platform and subsidiary tools such as CytoHubba were used to visualize the DEGs’ protein-protein interaction (PPI) network and identify hub genes, Furthermore our results are validated by using Single-cell RNA analysis. Results: Identification of 2524 genes exhibiting altered expression enriched in pathways including keratinization, epidermal cell differentiation, G alpha(s) signaling events, and biological process of cell proliferation and division, extracellular matrix (ECM) disassembly, and muscle function. Moreover, upregulation of hallmarks E2F targets, G2M checkpoints, and TNF signaling. CytoHubba revealed 20 hub genes that had a valuable influence on the progression of ESCC in these patients. Among these, the high expression levels of four genes, CDK1 MAD2L1, PLK1, and TOP2A, were associated with critical dependence for cell survival in ESCC cell lines, as indicated by CRISPR dependency scores, gene expression data, and cell line metadata. We also identify the molecules targeting these essential hub genes, among which GSK461364 is a promising inhibitor of PLK1, BMS265246, and Valrubicin inhibitors of CDK1 and TOP2A, respectively. Moreover, we identified that elevated expression of MMP9 is associated with worse overall survival in ESCC patients, which may serve as potential prognostic biomarker or therapeutic target for ESCC. The single-cell RNA analysis showed MMP9 is highly expressed in myeloid, fibroblast, and epithelial cells, but low in T cells, endothelial cells, and B cells. This suggests MMP9’s role in tumor progression and matrix remodeling, highlighting its potential as a prognostic marker and therapeutic target.

**Discussion:** Our study identified key hub genes in ESCC, assessing their potential as therapeutic targets and biomarkers through detailed expression and dependency analyses. Notably, MMP9 emerged as a significant prognostic marker with high expression correlating with poor survival, underscoring its potential for targeted therapy. These findings enhance our understanding of ESCC pathogenesis and highlight promising avenues for treatment.

## 1 Introduction

Cancer poses a significant threat to improving life expectancy as it remains one of the leading causes of global deaths (Bray et al., 2021). The preliminary data shows that it is the leading cause of mortality before the age of 70 in most countries worldwide. Since the last decade, cancer incidence and mortality rates have risen sharply around the globe, with 19.3 million new cases and 10 million cancer deaths worldwide in 2020 (Sung et al., 2021). It is estimated that more than 1,670 people will die of cancer every day in the United States by 2023 (Siegel et al., 2023). Among the malignancies of the gastrointestinal (GI) tract, esophageal cancer (EC) accounts for 3.2% of all the newly diagnosed cancer patients in the world, which is behind colorectal (10.2%) and stomach (5.7%) cancer (Bray et al., 2018). With an annual incidence of 572,000 & 47,000 and 509,000 & 42,000 deaths, EC is the 6th & 4th most common cause of cancer-related deaths worldwide (Siegel et al., 2018), and in India, respectively (Bray et al., 2018). Esophageal Squamous cell carcinoma (ESCC) and Esophageal adenocarcinoma (EAC) are the two most common kinds of EC, each with its own set of risk factors and pathological characteristics (Rustgi and El-Serag, 2014)^.^ While the ESCC arises from squamous epithelium, EAC is developed by intestinal metaplastic epithelial cells. However, ESCC continues to be the major type of cancer in many Asian countries, including India (80%) (Zhang et al., 2012). In contrast, the highest EAC instances were reported in Northern and Western Europe, North America, and Oceania (46% of the total global EAC cases (Rustgi and El-Serag, 2014).

The mechanism underlying ESCC is complicated and embraces a broad spectrum of hazards that contribute to its rapidly increasing incidence. There are two primary types of risk factors: inheritable and environmental factors. Environmental factors include alcohol and tobacco consumption, low vegetable and fruit intake, and low socioeconomic status (Sardana et al., 2018). As for EAC, almost all cases are complicated by precancerous lesions called Barrett’s esophagus, mainly derived from gastroesophageal reflux disease, a common condition throughout the human population (Coleman et al., 2018). Early-stage EC can be effectively treated with curative surgery, except for advanced cases with limited therapeutic strategies (Mocanu et al., 2015). Considering the differences in underlying biology, prognosis, patterns of recurrence, and response to currently available therapeis (Lieberman et al., 1995; Siewert and Ott, 2007; Bandla et al., 2012), these EAC and ESCC subtypes should not be treated similarly for drug development and therapeutic interventions (Cancer Genome Atlas Research Network et al., 2017), that may result in low therapy response and poor patient prognosis. Given its highly invasive nature and poor prognosis among gastrointestinal malignancies, a majority of people die from ESCC, placing a heavy burden on the international economy (Domper Arnal et al., 2015). The 5-year overall survival (OS) rate for patients with ESCC remains low at 10%–20% (Yu et al., 2016). In recent years, several molecular biomarkers with potential value in predicting the development of EC have been screened through high-throughput techniques, which can also help to reveal the molecular characteristics of cancer cells to predict the prognosis of patients (Yang et al., 2015). Despite these clues, the molecular mechanisms underlying ESCC development remain unclear, and there is a lack of effective molecular biomarkers to diagnose and predict the prognosis of ESCC.

Exploring pathological mechanisms of diseases based on bioinformatics theories has become an increasingly important and effective method (Hernández et al., 2020). With bioinformatics analysis, researchers can gain comprehensive knowledge regarding the studied diseases from molecular data. More crucially, it can provide novel insight leading to early diagnosis, definitive treatment, and survival prediction (Keerthikumar, 2017). Previous research in bioinformatics has yielded some achievements in ESCC knowledge, such as identifying associated genes and pathways and altered methylation associated with ESCC pathology (Peng et al., 2017; He et al., 2018). However, the analytical ability of ESCC has been limited due to insufficient sample size and a lack of in-depth evaluation of key genes that play a dominant role in the malignant development of ESCC.

This study analyzed ESCC RNAseq databases from TCGA and GTEX to identify DEGs between tumor and normal esophageal tissues through an integrated analysis of the datasets. The main biological functions of the identified DEGs were then explored by Gene Ontology (GO) annotation, Reactome pathways analysis, and msigDB cancer hallmark analysis. In addition, the protein-protein interaction (PPI) network of DEGs was used to identify the hub genes, which strongly influence pathogenesis. The core of our approach lies in network construction. By weaving a tapestry of interactions between DEGs, we identify “hub genes” occupying central positions and exerting potential control over cellular processes. To translate these insights into targeted therapy, we integrate data from DepMap, a comprehensive resource mapping genetic dependencies in cancer cells. This allows us to pinpoint hub genes with pronounced vulnerabilities, offering promising targets for drug intervention. Furthermore, by analyzing survival data within TCGA, we explore the predictive potential of hub genes. This investigation seeks to identify those whose expression levels correlate with patient survival, potentially acting as early indicators of disease progression or treatment response. By illuminating the interplay between dysregulated pathways, central hub genes, and their therapeutic and prognostic implications, this research strives to unveil a roadmap for improved ESCC management. Our findings can potentially guide the development of targeted therapies, enhance predictive accuracy, and ultimately translate into better outcomes for ESCC patients.

## 2 Methodology

### 2.1 Data collection and pre-processing

Gene expression data was retrieved from the UCSC Xena platform using the UCSC Xena Tools R package. Specifically, the RNA-Seq data sourced from the UCSC TOIL Recompute project was extracted and analyzed. RSEM software package, including TCGA, TARGET, and GTEX samples, was used to quantify the data. The expression data included a cohort of 753 samples (TCGA: 91 tumors, 11 matched normal, GTEX: 651 normal). The Clinical and survival data was obtained from cbioportal, incorporating relevant clinical variables from TCGA pan-cancer and TCGA firehose legacy datasets.

Before downstream analysis, the data was pre-processed to ensure the accuracy of the results. The pre-processing steps included the back-transformation of expected log-transformed count data from Xena Toil to potential zeros, refining of data to include normal samples from GTEX and primary tumor samples from TCGA, exclusion of non-coding genes and omission of data retrieved from the TARGET database. These data acquisition and pre-processing steps yielded a curated dataset for investigating gene expression, clinical characteristics, and survival outcomes in ESCC.

### 2.2 Identification of differentially expressed genes

DEGs were identified between cancer and normal samples using the limma package in R programming software (Ritchie et al., 2015). Significant DEGs were selected based on log2 fold change value ≥2 and *p*-value <0.05. Before analysis, potential outliers and genes lacking expression across all samples were filtered out to remove unwanted noise in the data. Normalization by upper quartile function was executed, ensuring robust scaling across varying library sizes. A contrast between gene expression profiles of TCGA samples with GTEX normal tissues was made using specialized Limma functions. Moreover, Voom transformation was applied before model fitting to address technical and biological variations in RNA-sequencing data. The data space was thus optimized, aligning variance closer to the mean.

### 2.3 Gene set enrichment analysis

The Gene Enrichment Analysis (GSEA) of high-confidence genes was done using cluster Profiler (Wu et al., 2021), and msigDB enrichment (Subramanian et al., 2005; Liberzon et al., 2015), in R programming software. Gene Ontology - Biological Process (GO-BP) terms and Reactome pathways were analyzed with a significant *p*-value cut off of <0.05. Using a cluster Profiler, the enrichment of significant DEGs was assessed through permutation tests. Furthermore, the msigDB was employed for hallmark gene set enrichment analysis on filtered DEGs, revealing broad functional themes relevant to the disease context.

### 2.4 Construction of protein-protein interaction network

A PPI of the genes enriched in GO-BP, Reactome pathways, and cancer hallmark gene sets was constructed using the STRING database v12.0 (Szklarczyk et al., 2011). The network of identifiers was visualized using Cytoscape Visualization Software version 3.10.1 (Shannon et al., 2003). In Cytoscape, the y file algorithm layout was applied to reveal interconnectivity patterns among interacting genes. The protein interaction data was combined with curated gene sets to form a valuable resource for exploring disease mechanisms and identifying therapeutic targets.

### 2.5 Identification of hub genes

The CytoHubba plugin within Cytoscape was employed to identify key genes within the PPI network (Shannon et al., 2003; Chin et al., 2014). Genes were selected based on the bottleneck algorithm embedded in CytoHubba (Przulj et al., 2004). Identifying such bottleneck genes can reveal master regulators and key drivers of cellular processes. Based on the bottleneck analysis, we selected the top 20 ranked hub genes for further investigation.

### 2.6 Cellular dependence and therapeutic targeting of hub genes

The top 20 identified hub genes within the PPI network were considered a starting point for developing novel therapeutic strategies against ESCC. Hubs were filtered out depending on ESCC survival and druggable dependencies, making them ideal targets for therapeutic intervention. This was done using the DepMap project, a comprehensive platform offering large-scale genetic perturbation data across multiple tumor types. Utilizing DepMap 23Q4 data (Tsherniak et al., 2017), we acquired critical datasets, including CRISPR dependency scores (“DepMap Public 23Q4+Score, Chronos”), Gene expression data, and Cell line metadata. We narrowed our analysis to specific cell lines related to ESCC and the previously identified hub genes. Subsequently, we accessed drug sensitivity data through the DepMap portal, specifically the “PRISM Repurposing Primary Screen,” which provides viability fractions for cancer cell lines treated with various drug doses. The drcR and ggplot R packages were utilized to fit the drug sensitivity data for hub genes in the ESCC context and visualize the fitted data, respectively. Hub genes exhibiting both essentiality and druggable dependencies stand as prime candidates for further preclinical and clinical studies, potentially leading to the development of novel therapeutic strategies.

### 2.7 Survival analysis of hub genes

To assess hub gene predictability for patient outcomes in ESCC, we conducted Cox regression analysis and Kaplan-Meier survival analysis. Cox Regression Analysis - Both univariate and multivariate Cox regression analyses were performed using the survival and ezcox packages in R programming software. The method measured the correlation between individual hub gene expression and overall survival in ESCC patients. In the multivariate analysis, age, stage, and gender served as covariates, controlling for potential confounders and enhancing the robustness of assessing the independent prognostic value of hub genes (Koletsi and Pandis, 2017).

Kaplan-Meier Survival Analysis - This method was implemented using the survminer package in R programming software to visualize the relationship between hub gene expression and patient survival times (Goel et al., 2010). Patients were categorized into high and low-expression groups based on the median expression level of each hub gene. Kaplan-Meier curves were generated for each group, depicting the proportion of patients surviving with increasing time. Log-rank tests with *p*-values <0.05 were used to evaluate the statistical significance of differences in survival between the high and low-expression groups.

### 2.8 Single cell analysis

Single-cell transcriptomics data was obtained from the GEO database through GEO query R packge (Davis and Meltzer, 2007). The data was sequenced using with 10x genomics platform comprising of 64 samples stained CD45 negative (non-immune cells) and CD45 positive (immune cells), out of which 60 were of ESCC patients and 4 adjacent normal tissues. The data was in the form of raw UMI count matrices of CD45 negative and positive with cell counts of 97,631 and 111,028, respectively. The UMI count matrices were processed through the Seurat R package, first low-quality cells were filtered out with low RNA counts and high mitochondrial gene content with a threshold of not <200 and >5. The data was normalized using the “Log Normalize” method using the Normalize Data function, and variable features were identified for downstream analysis using the Find Variable Features function (Leydesdorff and Bornmann, 2011). Further data was log-normalized and scaled using the scale Data function, and dimensionality reduction was performed through PCA (Sachin, 2015). The clustering was done with a graph-based method Louvain algorithm with a resolution 0.6 and neighbor identification through KNN, where K was set to 30 (Abdulla and Khasawneh, 2022). As per the reported cutoffs, the same number of clusters and their markers were identified as reported in the previous studies. The annotation was done using the available annotated data. The clustering results were visualized through the UMAP plot and gene expression of MMP9 among cells was visualized through violin plots.

## 3 Results

### 3.1 Overview of ESCC patient demographics and characteristics

This study drew upon a well-defined patient cohort from TCGA containing 93 individuals diagnosed with ESCC. Most patients were male (n = 81, 87%), with only a minority being female (n = 12, 13%). The median age at diagnosis was 57 years (interquartile range: 51–64). The cancer stages based on the American Joint Committee on Cancer (AJCC) staging system are distributed as follows - Stage I: 6.7%, Stage II: 59.0%, Stage III: 30.0%, and Stage IV: 4.4%. 3 Patients had unknown cancer stages. Patients had predominated Grade 2 (G2) histological grade (53%) followed by Grade 3 (G3): 23.0%, Grade 1 (G1): 15.0%, and Unknown Grade (GX): 9.7% (Table1). This distribution indicates a relatively balanced representation of early and advanced disease stages, allowing for comprehensive analysis across the disease spectrum. The GTEX dataset used had a total of 651 esophagus tissue samples. These samples were predominantly from the squamous region of the distal esophagus, at least 4 cm above the gastroesophageal junction. These esophageal samples represented normal tissues from healthy individuals. The donor demographics for the esophagus tissue collection in GTEX was skewed toward males. There were 415 male donors (64%) and 236 female donors (36%).

**TABLE 1:** Clinical characteristics of esophageal squamous cell carcinoma samples.

Characteristics				TCGA ESCC cohort	
				N = 93^*1*^	
	Stratification	Patients, (n %)		Stratification	Patients, (n %)
Age (Years)	51–64	57	Alcohol history	67	74%
Gender	Female	12 (13%)	Clinical grade	G1	14 (15%)
	Male	81 (87%)		G2	49 (53%)
Tumor site	Distal	39 (42%)		G3	21 (23%)
	Mid	41 (45%)		GX	9 (9·7%)
	Mid |Distal	4 (4·3%)	Clinical stage	I	6 (6·7%)
	Proximal	6 (6·5%)		II	53 (59%)
	Proximal| Mid	2 (2·2%)		III	27 (30%)
T stage	T1	7 (7·7%)		IV	4 (4·4%)
	T2	32 (35%)	N stage	N0	52 (57%)
	T3	48 (53%)		N1	29 (32%)
	T4	4 (4·4%)		N2	6 (6·6%)
M stage	M0	81 (90%)		N3	3 (3·3%)
	M1	3 (3·3%)		NX	1 (1·1%)
	M1A	1 (1·1%)	Race	Asian	44 (49%)
	MX	5 (5·6%)		Black or African American	5 (5·6%)
Survial Status	Deceased	32 (34%)		White	41 (46%)
	Living	61 (66%)			

^*1*^Median (IQR); n (%)

### 3.2 Identification of MMPs as a major regulator of ESCC

**FIGURE 1:**
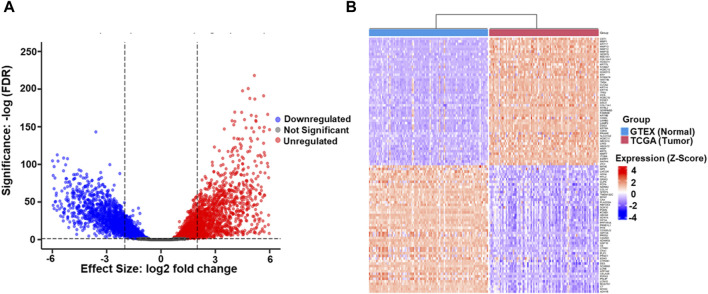
**(A)** The volcano plot illustrates the differential gene expression profile between TCGA-ESCC tumor samples and GTEX Esophageal normal tissue samples. Upregulated genes (n = 1,297), identified with a logFC (Logarithm of Fold Change) ≥ 2 and *p*-value <0.05, are highlighted in red, indicating higher expression in tumors. Downregulated genes (n = 1,227), meeting the criteria of LogFC ≤2 and *p*-value <0.05, are depicted in blue, signifying lower tumor expression. Genes with non-significant differential expression (n = 13,187) (*p*-value ≥0.05) are represented in grey. The LogFC threshold reflects the logarithmic ratio of expression levels between TCGA-ESCC tumors and GTEX normal tissue. In contrast, the significance threshold ensures statistical relevance in the identified gene expression changes **(B)**. The heatmap represents the top 100 DEGs, comprising 50 upregulated and 50 downregulated, identified between Esophageal Squamous Cell Carcinoma (ESCC) and GTEX datasets. Each row in the heatmap corresponds to an individual gene, while each column represents a distinct sample. The color spectrum employed in the heatmap signifies gene expression levels, with red denoting higher expression and blue indicating lower expression. The top 50 upregulated genes are depicted in shades of red, reflecting increased expression in ESCC compared to GTEX. Conversely, the top 50 downregulated genes are represented in shades of blue, signifying decreased expression in ESCC relative to GTEX.

After TCGA and GTEX data retrieval, the statistical criteria on *p* < 0.05 and 2 log FC ≥ 0.2 were utilized for further analysis. 15,711 DEGs between ESCC and normal Esophageal samples was identified, and rigorous filtering identified 2,524 genes exhibiting altered expression, including (1,227 downregulated genes and 1,297 upregulated DEGs). The remaining 13,187 genes did not show significant differential expression (Figure 1A). Further characterization of the DEGs revealed fascinating insights into potential drivers of ESCC pathogenesis. Notably, matrix metalloproteases (MMPs), known for their involvement in ECM remodeling and tumor invasion, were prominently featured among the top 50 most upregulated genes. This finding aligns with the aggressive nature of ESCC and emphasizes the potential involvement of MMPs in ESCC progression. Conversely, myosin family genes, essential for various cellular processes, including muscle contraction and cell movement, were enriched among the top 50 downregulated genes (Figure 1B). This downregulation suggests a potential disruption of cellular motility and contractility in ESCC, highlighting another potentially important aspect of ESCC biology.

### 3.3 Gene Ontology - Biological process enrichment analysis

**FIGURE 2:**
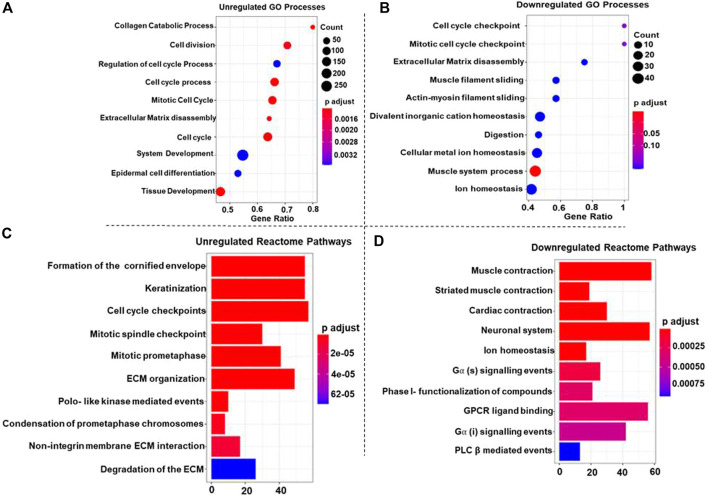
Dot plots representing the top 10 upregulated **(A)** and downregulated **(B)** Gene Ontology (GO) Biological Processes (GO-BP) associated with Esophageal Squamous Cell Carcinoma (ESCC). In each plot, individual dots represent specific GO-BP terms, and the dot size corresponds to the number of enriched genes within each biological process. The color gradient from blue to red indicates the significance level, with deeper red shades representing more significant enrichment and blue shades reflecting less significance. For the top 10 upregulated GO-BP terms **(A)**, the larger dots in warmer colors highlight biological processes with more enriched genes that play a role in ESCC. Conversely, in the top 10 downregulated GO-BP terms **(B)**, larger dots in warmer colors denote biological processes where many genes are downregulated in ESCC **(C, D)** Bar plots showcasing the top 10 upregulated **(C)** and downregulated **(D)** Reactome pathways associated with Esophageal Squamous Cell Carcinoma (ESCC). Each bar in the plot corresponds to a specific Reactome pathway, and the color gradient from blue to red signifies decreasing *p*-values, indicating increasing significance. In plot 2C, larger bars in warmer colors represent Reactome pathways with higher upregulated genes, showcasing key biological processes contributing to ESCC. Conversely, in plot 2D, larger bars in warmer colors denote Reactome pathways where many genes are downregulated in ESCC, providing insights into suppressed pathways.

GO-BP terms were performed on high-confidence genes with a *p*-value cut-off of <0.05 for statistical significance. The upregulated DEGs were significantly enriched in cell division, cell cycle process, cell cycle, mitotic cell cycle, ECM disassembly, and collagen catabolic process (Figure 2A). These findings highlight the enhanced proliferative capacity of ESCC cells, facilitating tumor progression and metastasis. Downregulated DEGs and the muscle system process were significantly enriched in muscle function. This suggests a potential loss of contractile capacity in ESCC cells, which could contribute to impaired esophageal motility (Figure 2B).

### 3.4 MSigDB cancer hallmark analysis highlights key pathways in ESCC progression

Reactome pathways were searched for up and downregulated DEGs with a *p*-value of <0.05 set as the statistical cut-off. The upregulated DEGs were significantly enriched in terms like the formation of the cornified envelope, cell cycle checkpoint, and mitotic spindle checkpoint pathways; additionally, pathways like Polo-like kinase mediated events, ECM organization, and keratinization emphasizing the aberrant cell cycle control in ESCC (Figure 2C). Furthermore, the downregulated DEGs were significantly enriched in muscle contraction, neuronal system, ion homeostasis” and G alpha (s) signaling events, GPCR ligand binding, and PLC β-mediated events pathways (Figure 2D), indicating disrupted ionic balance and signaling pathways, therefore these alterations might contribute to tumorigenesis and progression in ESCC.

### 3.5 msigDB cancer hallmark analysis

This analysis offers a broad overview of fundamental biological processes by identifying broad functional themes potentially impacting the disease context. Hallmarks like E2F Targets, G2M Checkpoint, Mitotic Spindle, and “TNFα Signaling were significantly upregulated. These findings align with the observed enrichment in cell cycle and proliferation-related pathways, further supporting the aggressive nature of ESCC. Also, downregulating hallmarks like Myogenesis and Adipogenesis suggest potential suppression of differentiation pathways in ESCC (Table 2).

**TABLE 2:** List of cancer hallmarks identified through enrichment analysis of differentially expressed genes in ESCC.

Cancer hallmarks	Pathway status	Net enrichment score	No. of enriched genes	*p-value*	*p-adjust*	*q-value*
G2M Checkpoint	Upregulated	3.94	56	1.0e-10	1.3e-09	8.1e-10
E2F Targets	Upregulated	3.72	45	1.0e-10	1.3e-09	8.1e-10
Mitotic Spindle	Upregulated	3.22	29	3.8e-09	3.7e-08	2.3e-08
TNFα Signaling	Upregulated	2.64	31	1.3e-05	9.9e-05	6.2e-05
MToRC1 Signaling	Upregulated	2.44	25	1.8e-04	1.2e-03	7.4e-04
P53 Pathway	Upregulated	2.30	12	3.3e-04	1.8e-03	1.1e-03
Interferon ɣ Response	Upregulated	2.27	24	4.6e-04	2.2e-03	1.4e-03
Estrogen response	Upregulated	2.06	33	1.1e-03	4.7e-03	2.9e-03
Glycolysis	Upregulated	2.03	29	5.2e-03	2.0e-02	1.2e-02
Inflammatory Response	Upregulated	1.97	28	5.8e-03	2.0e-02	1.3e-02
Interferon-α Response	Upregulated	1.84	13	1.2e-02	3.4e-02	2.1e-02
KRas Signaling	Upregulated	1.70	20	1.1e-02	3.2e-02	2.0e-02
Myogenesis	Downregulated	−3.87	59	1.0e-10	1.3e-09	8.1e-10
Adipogenesis	Downregulated	−1.84	10	1.0e-02	3.2e-02	2.0e-02

### 3.6 Construction of the PPI network

**FIGURE 3:**
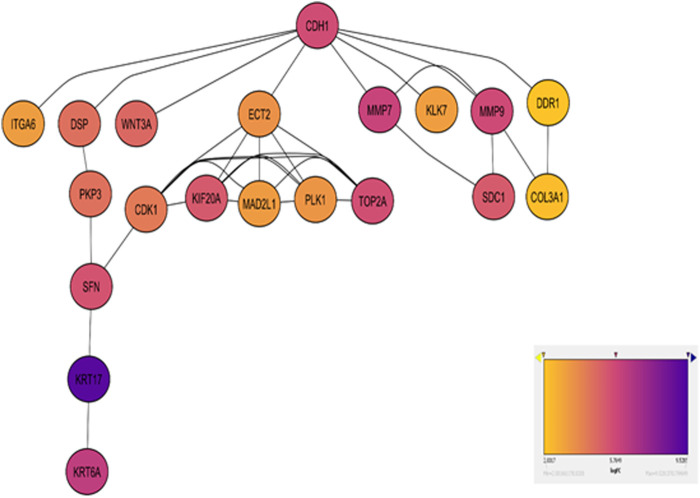
Network diagram visualizing the top enriched genes in Cell Cycle and ECM Degradation pathways. Red nodes highlight hub genes, with varying shades indicating log2 fold change, lighter shades for moderate changes, and darker shades for significant alterations. Gray edges depict gene interactions. A color gradient bar illustrates log2 fold change values.

Based on the functional enrichment analysis revealing prominent roles of cell cycle and ECM degradation in ESCC, further investigation focused on these pathways at the protein level. A curated set of 365 DEGs enriched in these pathways was employed to construct a PPI network. The PPI network exported by STRING comprised 365 nodes and 2,462 edges, which were visualized in Cytoscape (Figure 3). The modular interaction network was viewed as statistically enriched due to the enrichment *p*-value (*p* < 1.0e-16).

### 3.7 Identification and validation of differentially expressed hub genes

**FIGURE 4:**
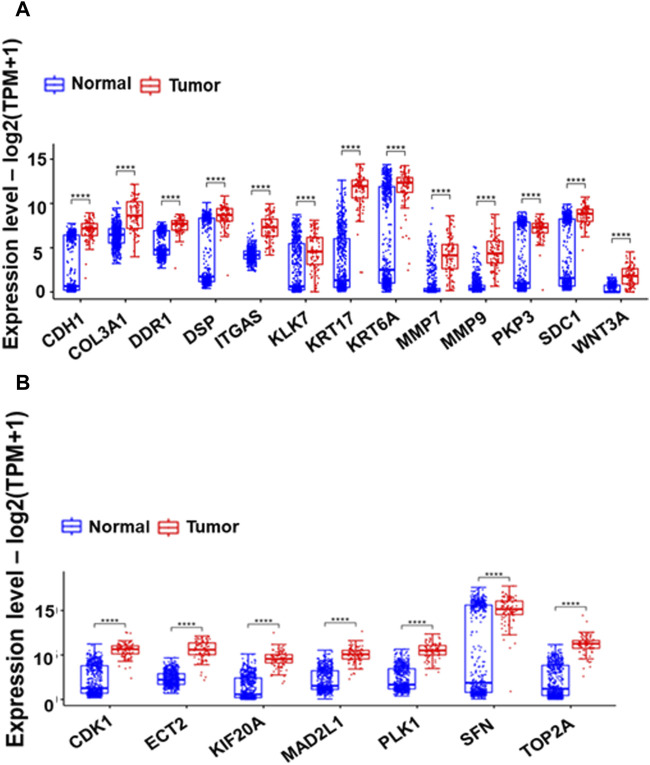
Box plots comparing the expression of 13 Extracellular Matrix genes **(A)** and seven cell cycle-related hub genes **(B)** between GTEX normal and Esophageal Squamous Cell Carcinoma (ESCC) tumor samples. The boxes depict the interquartile range of gene expression, with a horizontal line indicating the median. The presence of asterisks (*) signifies *p*-values less than 0.0001, highlighting significant differences in expression between normal and tumor samples.

The CytoHubba plugin identified the top 20 hub genes based on bottleneck algorithm ranking in the PPI network (Table 3), exhibiting high connectivity and potentially acting as critical regulators; these genes probably play crucial roles in the incidence and development of ESCC. These hub genes were categorized based on their functional association with either cell cycle or ECM degradation pathways. Interestingly, seven hub genes (CDK1, ECT2, KIF20A, MAD2L1, PLK1, SFN, and TOP2A) were linked to cell cycle regulation, highlighting the significance of this process in ESCC pathogenesis. Meanwhile, thirteen hub genes (CDH1, COL3A1, DSP, DDR1, ITGA6, KLK7, KRT17, KRT6A, MMP7, MMP9, PKP3, SDC1, and WNT3A) were associated with ECM degradation, reflecting the importance of this pathway in disease progression. To further validate the functional significance of the identified hub genes, the expression boxplots of the hub genes, based on TCGA and GTEX data, involved a comprehensive comparison of expression levels for all 20 hub genes between the tumor group (n = 91) and the normal group (n = 661). The results demonstrated that these hub genes were upregulated in the tumor group compared to the normal group. *P*-values (2.6E-49 to 1.4E-5), and the median log2 fold change (log2FC) between the two groups ranged from 1.8 to 10.6, providing strong evidence for their altered expression and substantial up- or downregulation of the hub genes in ESCC compared to normal tissues (Figure 4).

**TABLE 3:** Top 20 hub genes identified based on network analysis. The table categorizes these genes into two groups: 13 related to extracellular matrix remodelling and 7 related to cell cycle regulation.

Rank*	Hub gene	Gene name	Pathway	Involved in
1	CDH1	Cadherin-1	ECM	Cell-cell adhesion & gene transcription
2	ECT2	Epithelial cell transforming 2	Cell-Cycle	Cell proliferation & migration
3	SFN	Stratifin	Cell-Cycle	Cell differentiation & apoptosis
4	MMP9	MMP9	ECM	ECM degradation & cell invasion
5	KRT17	Keratin 17	ECM	Epithelial cell structure & function
6	MMP7	MMP 7	ECM	ECM degradation & cell invasion
7	TOP2A	Topoisomerase II α	Cell-Cycle	DNA replication & transcription
8	MAD2L1	MAD2 like 1	Cell-Cycle	Cell cycle regulation & spindle checkpoint control
9	PKP3	Plakophilin 3	ECM	Desmosome formation and cell-cell adhesion
10	ITGA6	Integrin α6	ECM	Cell-matrix adhesion and cell signaling
11	CDK1	Cyclin-dependent kinase 1	Cell-Cycle	Cell cycle regulation & mitosis
12	DDR1	Discoidin domain receptor 1	ECM	Cell adhesion and migration
13	COL3A1	Collagen type III α 1 chain	ECM	ECM structure and function
14	KRT6A	Keratin 6A	ECM	Epithelial cell structure and function
15	WNT3A	Wingless-type MMTV integration site family member 3A	ECM	Cell proliferation, differentiation & migration
16	KLK7	Kallikrein-related peptidase 7	ECM	Proteolysis and cell signaling
17	SDC1	Syndecan 1	ECM	Cell adhesion, cell signaling, and ECM organization
18	PLK1	Polo-like kinase 1	Cell-Cycle	Cell cycle regulation and mitosis
19	KIF20A	Kinesin family member 20A	Cell-Cycle	Microtubule-based intracellular transport
20	DSP	Desmoplakin	ECM	Desmosome formation and cell-cell adhesion

^**^Rank is based on The Bottleneck Algorithm^*^

ECM, extracellular matrix; MMP, matrix metalloprotease

### 3.8 Hub genes as potential therapeutic targets

**FIGURE 5:**
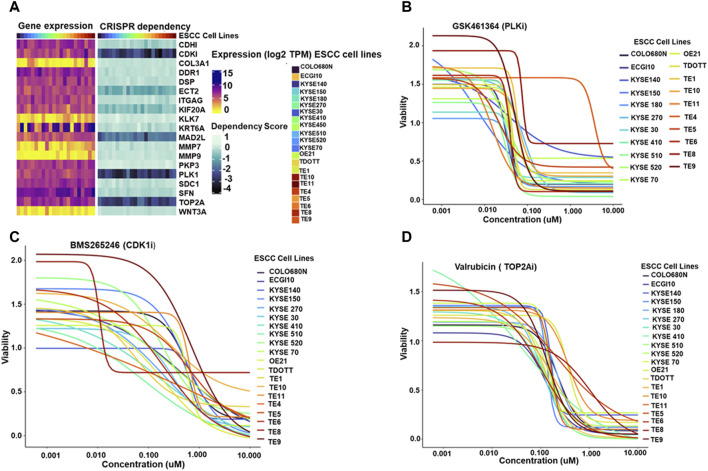
**(A)** Paired heatmaps depicting gene expression of hub genes and CRISPR dependency scores across 22 Esophageal Squamous Cell Carcinoma (ESCC) cell lines. The color gradients in the heatmaps provide a quick reference for gene expression levels and cellular dependence, with lighter shades representing lower expression or dependency and darker shades indicating higher levels **(B–D)**. Dose-response plots illustrating the effect of PLK1 inhibitor GSK461364 (5B), CDK1/2 inhibitor BMS265246 **(C)**, and TOP2A inhibitor Valrubicin **(D)** on ESCC cell line viability. The X-axis represents the concentration of the respective inhibitors-PLK1 inhibitor GSK461364, CDK1/2 inhibitor BMS265246, and TOP2A inhibitor Valrubicin- while the Y-axis illustrates the corresponding viability fraction of ESCC cells. These graphs succinctly portray the relationship between increasing drug concentrations and the resultant viability of ESCC cell lines, offering a visual representation of the potential effectiveness of each inhibitor in influencing cell viability in the context of ESCC.

To further explore the potential therapeutic implications of the identified hub genes, their dependency in ESCC cell lines was investigated (Figure 5A). displays the heatmap depicting the expression of hub genes across 22 ESCC cell lines.

The study investigated the expression of four essential hub genes—CDK1, MAD2L1, PLK1, and TOP2A-as critical dependencies for cell survival in ESCC cell lines. These genes were identified using CRISPR dependency scores, all ≤ −1, indicating a strong dependence on these genes for cell viability.

Next, the study identified available molecules that could target these essential hub genes. Among the screened compounds, GSK461364 emerged as a promising PLK1 inhibitor. BMS265246 and Valrubicin were identified as effective inhibitors of CDK1 and TOP2A, respectively. Unfortunately, no readily available inhibitor was found for MAD2L1.

Dose-response plots were generated to illustrate the effect of these inhibitors on ESCC cell lines. The majority of the ESCC cell lines demonstrated sensitivity to GSK461364 and Valrubicin at sub-micromolar concentrations. BMS265246 also showed efficacy, with sensitivity observed at micromolar concentrations (Figures 5B–D). These findings highlight the potential of targeting essential hub genes, particularly PLK1, TOP2A, and potentially CDK1, for therapeutic intervention in ESCC. GSK461364 and Valrubicin appear promising candidates for further investigation, warranting further studies to establish their specific efficacy and safety in ESCC treatment.

### 3.9 Prognostic analysis of hub genes in ESCC reveals MMP9 as a potential biomarker

**FIGURE 6:**
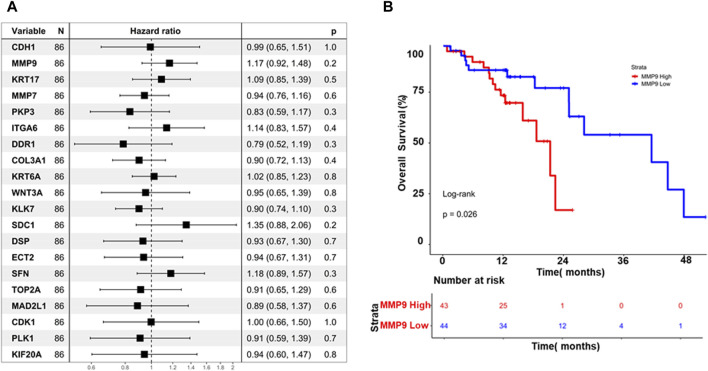
**(A)** Displays a forest plot summarizing the impact of hub gene expression on overall survival in Esophageal Squamous Cell Carcinoma (ESCC), using hazard ratios (HR). Each line represents an individual hub gene, with position indicating HR and confidence intervals **(B)** Kaplan-Meier curves illustrate the difference in overall survival between high and low MMP9 expression groups over time. These visuals efficiently convey the collective influence of hub genes on survival and highlight the prognostic significance of MMP9 expression in ESCC.

The association between hub genes and prognosis in ESCC was explored in this study, employing a two-pronged approach: cox regression analysis and Kaplan-Meier survival analysis. The Cox regression analysis investigated that none of the hub genes demonstrated a statistically significant association with overall survival (*p* > 0.05), as illustrated in Figure 6A. This finding suggests that hub genes may not possess sufficient prognostic power in ESCC. While the study further evaluated their impact on survival rates through Kaplan-Meier analysis, interestingly, MMP9 emerged as the sole gene exhibiting a significant log-rank *p*-value (*p* = 0.026), as depicted in (Figure 6B). Patients with high MMP9 expression displayed worse overall survival than those with low expression. This finding highlights the potential prognostic value of MMP9 in ESCC, independent of the lack of individual significance observed in the Cox regression analysis.

### 3.10 Validation using single-cell RNA analysis

**FIGURE 7:**
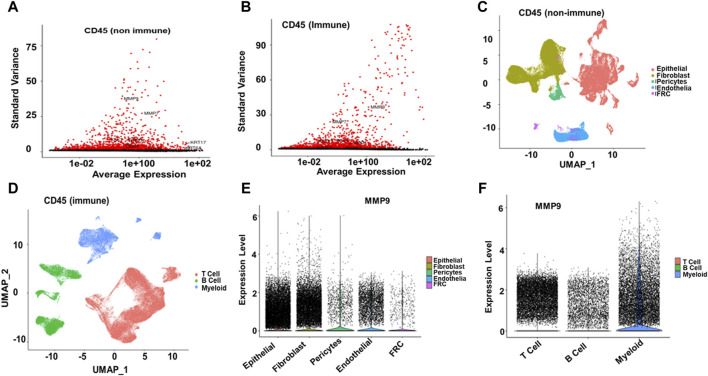
Single-cell RNA Seq analysis **(A, B)** Among CD45-negative cells, MMP9, MMP7, KRT17, KLK7, TOP2A, CDK1, and KRT6A were identified as highly variable features. In contrast, in EC samples, MMP9, MMP7, PLK1, KRT17, and SDC1 exhibited significant variability among CD45-positive cells **(C, D)** Cell annotation identified eight main cell types at a resolution of 0.6, resulting in the same clusters reported with cell markers. The annotated cell types are as follows: CD45-negative epithelial cells (44,730), fibroblasts (37,213), endothelial cells (11,267), pericytes (3,102), and fibroblastic reticular cells (1,319). For CD45-positive cells, the annotated types are T cells (69,278), B cells (22,477), and myeloid cells (19,273) **(E, F)**. MMP9 was highly expressed in myeloid cells, fibroblasts, and epithelial cells, had low expression in T and endothelial cells, and was negligible in pericytes, fibroblastic reticular cells, and B cells.

Single-cell RNA analysis revealed that MMP9, MMP7, KRT17, KLK7, TOP2A, CDK1, and KRT6A were highly variable features among the CD45-negative cells. In contrast, MMP9, MMP7, PLK1, KRT17, and SDC1 showed variability in CD45-positive cells in ESCC samples (Figures 7A, B). Further cell annotation identified eight main cell types, with a resolution of 0.6, resulting in clusters that aligned with known cell markers. Consequently, we annotated the CD45-negative cells as follows: epithelial cells (44,730 cells), fibroblasts (37,213 cells), endothelial cells (11,267 cells), pericytes (3,102 cells), and fibroblastic reticular cells (1,319 cells). The CD45-positive cells were categorized into T cells (69,278 cells), B cells (22,477 cells), and myeloid cells (19,273 cells) (Figures 7C, D). MMP9 was found to be differentially expressed across various cell types in ESCC. Specifically, it was highly expressed in myeloid cells, fibroblasts, and epithelial cells. In contrast, MMP9 exhibited low expression in T cells and endothelial cells and negligible expression in pericytes, fibroblastic reticular cells, Figures 7C, D and B cells (Figures 7E–F).

## 4 Discussion

ESCC is a major global health challenge with multifactorial etiology, including genetic and environmental components. Efforts to detect inchoate changes have attenuated the development of cancer and have even had some success in prevention (Alsop and Sharma, 2016). Therefore, seeking predictive indicators and therapeutic markers for ESCC is vital. In our research, 15,711 DEGs were screened via R programming software. Then, GO-BP function enrichment analysis, Reactome pathway enrichment analysis, and msigDB cancer hallmark analysis were applied to filter the DEGs. A PPI network was constructed using STRING to obtain hub genes likely central to the ESCA pathological process. Most previous studies in this field stopped at this point, resulting in limited clinical usefulness. Therefore, we implemented further analysis targeting the crucial hub genes identified previously in our study. We focused on assessing screened hub genes with greater possibility as new therapeutic targets for treating ESCC. We conducted an expression analysis of these hub genes, explored their potential therapeutic implications and dependency in ESCC cell lines, identified inhibitory molecules targeting these essential hub genes, and conducted the prognostic analysis. These analyses showed the potential of these hub genes as biomarkers for appraisal in therapy.

This study conducted a detailed analysis of a patient cohort comprising 93 individuals diagnosed with ESCC, sourced from TCGA. The cohort had a significant gender disparity, with 87% of the patients being male (n = 81) and only 13% female (n = 12). This male predominance is noteworthy and may warrant further investigation into gender-specific risk factors or differences in disease progression. The median age at diagnosis for this cohort was 57 years, with an interquartile range of 51–64 years. This suggests that ESCC tends to be diagnosed in middle-aged to older adults, highlighting the importance of age-related screening and early detection strategies. Moreover, the distribution of cancer stages, according to the AJCC staging system, revealed that Stage II was the most prevalent, affecting 59.0% of patients.

Additionally, most patients had Grade 2 (G2) histological tumors, accounting for 53% of the cases, which was higher than the other histological grades. Furthermore, the characterization of the DEGs revealed fascinating insights into potential drivers of ESCC pathogenesis. Notably, MMPs, known for their involvement in ECM remodeling and tumor invasion, were prominently featured among the top 50 most upregulated genes. Previous studies have shown that MMP9 was associated with ESCC cell migration and invasion, and the Stat3 signaling pathway controlled its expression *in vitro* (Tsukamoto et al., 2023). This finding aligns with the aggressive nature of ESCC and emphasizes the potential involvement of MMPs in ESCC progression.

Moreover, upregulation of hallmarks like “E2F Targets,” “G2M Checkpoint,” “Mitotic Spindle,” and “TNFα Signaling.” These findings align with the observed enrichment in cell cycle and proliferation-related pathways, further supporting the aggressive nature of ESCC. Furthermore, hallmarks like “Myogenesis” and “Adipogenesis” suggest potential suppression of differentiation pathways in ESCC. Notably, the upregulation of interferon and TNF signaling pathways suggests the presence of a “hot” tumor phenotype in some ESCC subtypes. Furthermore, the enrichment of DEGs in cell proliferation and division, including “collagen catabolic process,” “cell division,” “regulation of cell cycle process,” and “mitotic cell cycle,” and ECM disassembly, suggested that multitudes of DEGs were closely associated with nuclear activities, like cell division. Cell division as a functional category includes mechanisms to properly orient and position the mitotic spindle, which is essential because incorrect activity related to the spindle contributes to disease, even carcinogenesis (Canman and Cabernard, 2018; López-Lázaro, 2018); these findings highlight the enhanced proliferative capacity of ESCC cells. Also, the most abundant matrix protein polymers are collagens, which increase tumor tissue stiffness, regulate tumor immunity, and promote metastasis (Discher et al., 2017; Yamauchi et al., 2018). Therefore, enrichment in terms of collagen catabolism is of great significance in the development of ESCC.

Moreover, reactome pathway enrichment analysis revealed that upregulated DEGs were also enriched in keratinization pathways, cornified envelope formation, and epidermal cell differentiation. According to previous literature, enrichment analysis showed that the keratinization process was focused, and a series of genes were related to the development of esophageal cancer (Song et al., 2023). In one study, keratinization is accompanied by apoptosis and is ultimately associated with tumor progression in patients with ESCC (Ohbu et al., 1995). Also, enrichment in cell cycle checkpoint and mitotic spindle checkpoint pathways further emphasizes the aberrant cell cycle control in ESCC. Therefore, these alterations might contribute to tumorigenesis and progression in ESCC.

Furthermore, the top 20 hub proteins are closely associated with the cell cycle, tumorigenesis, and ECM degradation (Gobin et al., 2019; Liu et al., 2019; Quintero-Fabián et al., 2019; Wu et al., 2019; Xie et al., 2020; Pandey et al., 2021; Hosseini and Nemati, 2023). To further explore the potential therapeutic implications of the identified hub genes, their dependency in ESCC cell lines was investigated. Among 20 selected hub genes, the expression of four essential hub genes, CDK1, MAD2L1, PLK1, and TOP2A, is critical for cell survival in ESCC cell lines. Previous studies showed that low expression of CDK1 was associated with a worse relapse‐free survival rate in ESCC patients (Dong et al., 2018). TOP2A has been reported to be a sensitive and specific marker of active proliferating cells, indicating its importance in cancer research (D’arcy and Gabrielli, 2017). A large-scale retrospective study has demonstrated that TOP2A high expression is associated with poor differentiation and neural invasion of esophageal cancer and is also an independent risk factor affecting the prognosis of esophageal cancer (Xu et al., 2015). Also, The expression of PLK1 is upregulated in various tumors, which is often associated with poor prognosis, including ESCC (Takai et al., 2005; Feng et al., 2009).

Next, our study focused on identifying available molecules targeting these essential hub genes. GSK461364 emerged as a promising PLK1 inhibitor, while BMS265246 and Valrubicin were identified as inhibitors of CDK1 and TOP2A, respectively. Unfortunately, no readily available inhibitor was found for MAD2L1. These findings highlight the potential of targeting essential hub genes, particularly PLK1, TOP2A, and potentially CDK1, for therapeutic intervention in ESCC. GSK461364 and Valrubicin appear promising candidates for further investigation, warranting further studies to establish their specific efficacy and safety in ESCC treatment. The Cox regression analysis finding suggests that hub genes may not possess sufficient prognostic power in ESCC. While the study further evaluated their impact on survival rates through Kaplan-Meier analysis, interestingly, MMP9 emerged as the sole gene exhibiting a significant log-rank *p*-value (*p* = 0.026). Patients with high MMP9 expression displayed worse overall survival than those with low expression. This finding highlights the potential prognostic value of MMP9 in ESCC, independent of the lack of individual significance observed in the Cox regression analysis.

In our single-cell RNA analysis, we observed that MMP9, among other markers, was highly variable across different cell types in ESCC samples, particularly in CD45 negative epithelial cells and CD45 positive myeloid cells. Notably, MMP9 exhibited differential expression patterns, being highly expressed in myeloid, fibroblast, and epithelial cells while showing low or negligible expression in T cells, endothelial cells, pericytes, fibroblastic reticular cells, and B cells. The high expression in myeloid cells suggests significant involvement in immune-mediated processes and inflammation within the TME. This could contribute to creating pro-tumorigenic inflammatory conditions. Elevated MMP9 expression facilitates ECM remodeling in fibroblasts and epithelial cells, potentially enhancing tumor invasion and metastasis. The presence of MMP9 in these stromal and cancer cells may promote tissue restructuring, angiogenesis, and cancer cell migration. The lower expression in T cells might indicate a secondary role in adaptive immune responses, possibly through cytokine-mediated effects. The minimal expression in endothelial cells, pericytes, fibroblastic reticular cells, and B cells suggests that MMP9’s direct effects on vascular remodeling and lymphoid tissue architecture are limited. This expression pattern aligns with the bulk RNA-seq finding that underlines higher MMP9 levels correlate with worse overall survival in EC patients, highlighting its potential as a prognostic marker and therapeutic target, particularly through its actions in myeloid, fibroblast, and epithelial cell populations.

## 5 Conclusion and limitation of study

We identified 7,055 differentially expressed genes (DEGs) in ESCC, including 3,312 downregulated and 3,743 upregulated genes and 20 hub genes. Four critical genes- CDK1, MAD2L1, PLK1, and TOP2A are essential for ESCC cell survival, suggesting their potential as prognostic biomarkers or therapeutic targets. GSK461364 is a promising PLK1 inhibitor, while BMS265246 and Valrubicin target CDK1 and TOP2A, respectively. DEGs are involved in key processes and pathways like cell cycle, ECM disassembly, collagen catabolism, and several signaling events. Hallmarks such as E2F Targets, G2M Checkpoint, and TNFα Signaling are upregulated, while Myogenesis and Adipogenesis are downregulated, highlighting the aggressive nature of ESCC and potential suppression of differentiation pathways. Moreover, MMP9, among other markers, was highly variable across different cell types in ESCC samples, and its high expression of MMP9 correlates with poor survival.

These findings provide insights for future predictive biomarker exploration and targeted therapies in ESCC. While we have initiated validation, further experimental studies are required to verify our results. Despite this limitation, our data provide valuable insights to guide future exploration of predictive biomarkers and molecular-targeted therapy for ESCC.

## Data Availability

The datasets presented in this study can be found in online repositories. The names of the repository/repositories and accession number(s) can be found in the article/supplementary material.
